# Review of 2,4-dichlorophenoxyacetic acid (2,4-D) biomonitoring and epidemiology

**DOI:** 10.3109/10408444.2012.710576

**Published:** 2012-08-10

**Authors:** Carol J. Burns, Gerard M. H. Swaen

**Affiliations:** 1 Department of Epidemiology, The Dow Chemical Company, Midland, MI, USA and; 2 Department of Epidemiology, Dow Benelux, B.V., Terneuzen, The Netherlands

**Keywords:** 2,4-D, herbicide, non-Hodgkin lymphoma, cancer, biomonitoring

## Abstract

A qualitative review of the epidemiological literature on the herbicide 2,4-dichlorophenoxyacetic acid (2,4-D) and health after 2001 is presented. In order to compare the exposure of the general population, bystanders and occupational groups, their urinary levels were also reviewed. In the general population, 2,4-D exposure is at or near the level of detection (LOD). Among individuals with indirect exposure, i.e. bystanders, the urinary 2,4-D levels were also very low except in individuals with opportunity for direct contact with the herbicide. Occupational exposure, where exposure was highest, was positively correlated with behaviors related to the mixing, loading and applying process and use of personal protection. Information from biomonitoring studies increases our understanding of the validity of the exposure estimates used in epidemiology studies. The 2,4-D epidemiology literature after 2001 is broad and includes studies of cancer, reproductive toxicity, genotoxicity, and neurotoxicity. In general, a few publications have reported statistically significant associations. However, most lack precision and the results are not replicated in other independent studies. In the context of biomonitoring, the epidemiology data give no convincing or consistent evidence for any chronic adverse effect of 2,4-D in humans.

## Introduction

While most regulatory agencies heavily rely on toxicological data, the results of epidemiology studies are becoming more and more important in this area. For this reason and for public health purposes, in general, a critical review of the epidemiologic literature on crop protection products is of increasing value. The herbicide, 2,4-dichlorophenoxyacetic acid (2,4-D), is an example of a pesticide for which the epidemiology data are continually reviewed and debated. Recently re-registered for use by Health Canada (2008), and the US Environmental Protection Agency, EPA (2005), 2,4-D is currently being re-evaluated by the European Union. A past review by Gandhi et al. (2000) emphasized the inconsistent nature of the studies up to 2000 that precluded drawing any conclusion on carcinogenicity in humans. Reviewing the literature through 2001, Garabrant and Philbert (2002) observed more strongly that the epidemiology data “provide scant evidence that supports a conclusion that exposure to 2,4-D is associated with STS [soft tissue sarcoma], NHL [non-Hodgkin lymphoma] or HD [Hodgkin's Disease].” Since no qualitative review of the 2,4-D epidemiology literature since 2001 has been published we present an update in this paper.

The herbicide, 2,4-D, has been registered for use since the 1940s. As a selective herbicide, 2,4-D is used to control broadleaf weeds in a variety of settings from crops, rights-of-way, lawns, forests to aquatic settings. In aerobic environments, 2,4-D degrades rapidly from 2 to 13 days (Wilson et al., 1997). In humans, 2,4-D is excreted unmetabolized in urine with a half-life of 10 to 33 hours, an average of 17.7 hours (CDC, 2009; Sauerhof et al., 1977). 2,4-D is cleared into urine of both animals and humans by a saturable organic anion transporter, OAT-1; toxicity of 2,4-D in rodents is typically limited to dose levels that saturate renal clearance (>50 mg/kg/day). Toxicity observed in rodents at doses above renal saturation is generally not regarded as relevant to human health risk (Timchalk, 2004; EPA, 2005). Chronic toxicity of 2,4-D has been tested in laboratory animals at a wide range of dose levels (Garabrant & Philbert, 2002; EPA, 2005). Studies in both rats and mice have shown no carcinogenic effect of 2,4-D and the US EPA classifes 2,4-D as a Group D chemical (not classifable as to human carcino-genicity). In two-generation reproduction studies there were no effects seen on fertility indices at doses up to and including 72 mg/kg/day. Reduced bodyweight gain has been seen at the higher doses (generally above the level at which renal excretion is saturated), with reduced food intake usually seen in parallel. Experimental in vitro and in vivo animal studies show no genotoxic potential for 2,4-D. Neurotoxicity studies showed aberrations in locomotion and open feld behavior at high doses saturating renal clearance but no histopathological findings in neural tissues. The neurotoxic signs were reversible and were at doses that exceeded general toxicity. As one of the most widely used herbicides in the world, 2,4-D continues to be one of the most studied pesticides, both in animals and in humans.

The purpose of this review is to provide a qualitative review of the human biomonitoring and epidemiology data since the summaries by Garabrant and Philbert (2002) and Munro et al. (1992). The biomonitoring data on urine samples include publications since 1991, as they provide the most reliable information on human exposure. To our knowledge these data have not yet been comprehensively reviewed. Some exposure studies were conducted in the 1980s on commercial applicators to turf, forestry and crops (Kolmodin-Hedman & Erne, 1980; Kolmodin-Hedman et al., 1983; Yeary, 1986). These studies will not be included since technical changes, specifcally improvements in analytical methods, have made these early studies less comparable to recent ones.

## Methods

### Scope

In 1986, the International Agency for Cancer Research (IARC) classified the chlorophenoxy herbicides as a Group 2B (possible) carcinogen (IARC, 1986). Tis monograph evaluated the group, which in addition to 2,4-D, includes other phenoxies such as MCPA and 2,4,5-trichlorophenoxyacetic acid (2,4,5-T) and related impurities of polychlorinated dioxins. Notably, IARC has not identified 2,4-D, *per se*, as a possible carcinogen. IARC sponsored an occupational cohort mortality study of 21,863 workers across 12 countries exposed to phenoxy herbicides. In their 1997 report, Kogevinas and colleagues stratifed the workers by those exposed to the contaminant 2,3,7,8-tetrachlorodibenzo-p-dioxin (TCDD) and other chlorinated dioxins and workers unexposed to dioxins (Kogevinas et al., 1997). These investigators noted that the dioxin contaminants have similar mechanisms of action and toxicities whereas the herbicides such as 2,4-D were not known to contain TCDD. In their review, Garabrant and Philbert (2002) also designated cohorts with co-exposure to TCDD. The current review of human epidemiology and 2,4-D will use a similar approach. Where possible, results specific to 2,4-D will be presented and reviewed.

### Interpretation

Tere are many approaches to compiling and reviewing a body of literature. The criteria or guidelines as proposed by Bradford Hill provide useful components to assess data. Swaen and Amelsvoort (2009) proposed a quantitative approach to empirically consider the Hill criteria. They tested their approach using the agents classified as Class 1 or 2A carcinogens by the IARC and observed that in addition to the experimental evidence, the criteria of strength of association and consistency had the greatest weight. In the current review, we did not evaluate the animal or experimental data. Summaries are available from government regulatory agencies such as the US EPA and Health Canada (US EPA, 2005; Health Canada, 2008). A strong association was considered to be a risk estimate or odds ratio greater than 2. We looked for consistency or replication of statistically significant results both within the same study and in independent studies. For example, results were considered to be internally inconsistent if exploratory analyses of all subjects were statistically signifcantly (*p* < 0.05 or the upper and lower bound of the confidence limits excluded the null value) but analyses by dose level were not statistically significant. We looked for replication of statistically significant results of specific outcomes to be reported in more than one study to meet the guideline for external consistency.

Biomonitoring data were also considered to be informative. Limited data on exposure are generally considered to be the most critical component of epidemiology studies on pesticide exposure and potential health outcomes. Good quality biomonitoring data can significantly strengthen this shortcoming and can even provide a reliable metric for internal exposure. The biomonitoring data also provide an excellent opportunity to put the exposure to 2,4-D of the various human populations into its proper perspective. Therefore we have included a specific review of the available biomonitoring data on 2,4-D. These data were organized by opportunity for exposure (general population, bystander and occupational) to be in line with populations that are included in epidemiology studies and this approach is similar to that taken by regulators and risk assessors.

### Search strategy

As part of a larger project to report the toxicology of 2,4-D, a systematic literature search using the terms “2,4-dichlorophenoxyacetic acid” and the chemical abstract service registration number, “94-75-7” was conducted on the databases BIOSYS, Pascal, Agricola, Chemical Safety News base, ELSEVIER, and CA SEARCH during the years 2000 to 5 January 2012. Another search contained more search terms that included “epidemiology,” “human,” “occupation,” “workers,” “women,” “men,” and “farmers.” More than 12,000 references were identified. The abstracts were manually screened by topic and relevance. The studies relevant to human, epidemiology and biomonitoring were selected for this review. Although English language was not a criterion there were no relevant studies identified that needed translation into English. In addition, papers were reviewed when cited as part of other studies and publications.

### Inclusion and exclusion

Studies that evaluated human health effects following exposure to herbicides in general were not considered. There are many chemicals used to control vegetation. The US EPA reported that in 2001, 2,4-D use in the United States was ranked third to herbicides glyphosate and atrazine (US EPA, 2012). A survey for more than 35,000 pesticides applicators as part of the Agricultural Health Study observed that pesticides use varies by residence and commodity (Alavanja et al., 1999). Without further information from selected studies, we concluded that we would not attribute to 2,4-D a reported association of “herbicides” and a health effect. For example, we excluded the cancer mortality and illness and injury studies of herbicide applicators (Wesseling et al., 2001; Swaen et al., 2004) for this reason. We excluded other studies for which the results were not specific to 2,4-D because they implied exposure from residence (Schreinemachers, 2003) or occupation (Garaj-Vrhovac & Zeljezic, 2000, 2001, 2002; Gómez-Arroyo et al., 2000; Zeljezic & Garaj-Vrhovac, 2002; Martínez-Valenzuela et al., 2009; Remor et al., 2009).

Included were results for phenoxy herbicides and 2,4-D. In publications that reported analyses for all pesticides combined as well as individual exposures, only the results specifically addressing 2,4-D were reviewed.

## Review of urinary biomonitoring

### General population

The largest general population studies of 2,4-D urinary levels in children and adults are those conducted by the US Centers for Disease Control and Prevention (CDC) (2009) and Health Canada (2010). With 2412 and 5480 subjects, respectively, both studies were designed to be representative of the national population (Table 1). Neither study detected 2,4-D at the 50th percentile (less than 1 parts per billion, ppb). These findings are similar to an earlier study by CDC (Hill et al., 1995). The US EPA's study of children and adults in North Carolina and Ohio (Morgan et al., 2008) and a study of children in Tailand (Panuwet et al., 2009), both observed exposure levels to be at or below 1 ppb. The maximum values shown in Table 1 ranged from 2 to 37 ppb. These studies demonstrate that background exposure to 2,4-D is low to undetectable in most populations. Certain individuals had the opportunity for exposure as evidenced by the maximum urinary levels. However, few of the studies collected data relevant to diet or activities to determine how the contact or exposure to 2,4-D occurred.

**Table 1:** General population, no a priori exposure, in µg/L (ppb).

					Selected percentiles				
						
Source	Group	Number of subjects	LOD	% < LOD	25th	GM (AM)	50th	75th	Max
Centers for Disease Control and Prevention (CDC) (2009)	US 2001-2 population, Ages 6–59	2413	0.2	NR	<LOD	NC	<LOD	<1	95th = 1.3
Health Canada (2010)	Canadian population, Ages 6–79	5480	NR	95	<LOD	NC	<LOD	<LOD	95th < LOD
Hill et al. (1995)	US adults	983	1	88	<LOD	(<1)	<LOD	<LOD	37
Morgan et al. (2008)	NC adults	66	0.2	14	<1	NR	<1	1	5
Morgan et al. (2008)	OH adults	69	0.2	13	<1	NR	1	1	8
Morgan et al. (2008)	NC children	66	0.2	12	<1	NR	<1	1	3
Morgan et al. (2008)	OH children	69	0.2	3	<1	NR	1	2	13
Panuwet et al. (2009)	Tailand children	207	0.5	83	NR	<1 (<1)	<1	NR	3

AM, arithmetic mean; GM, geometric mean; LOD, limit of detection; NC, not calculated; NR, not reported.

### Bystanders, indirect exposure

There are a number of urinary measurements collected from groups of individuals considered to be bystanders (Table 2). These individuals do not mix, load or apply 2,4-D, but may have the opportunity for indirect exposure over and above the general population. Examples include spouses and children of applicators, as well as applicators who apply other herbicides. The largest study reported that urinary 2,4-D was detected (LOD = 0.2 ppb) in all but 2% of 196 farm workers (Arcury et al., 2010) although actual urinary levels were not reported. Another large study of 125 spouses of farmer applicators observed that most of the individuals were below 1 ppb (Arbuckle & Ritter, 2005). Other studies of women reported the geometric mean 2,4-D level to be 1 pbb or less (Harris et al., 1992; Cooper et al., 2001; Alexander et al., 2007). The monitoring levels of bystander children were slightly higher than the parents (Arbuckle et al., 2004; Alexander et al., 2007; Arcury et al., 2007). However, a few children and spouses were involved in the application process, invalidating their classification as “bystanders.” The remaining bystander studies collected urine from crop applicators before the application (Alexander et al., 2007) or from crop applicators that applied other products (Arbuckle et al., 2002; Curwin et al., 2005; Panuwet et al., 2008; Bakke et al., 2009) and licensed applicators in Minnesota (Garry et al., 2001). In general, the central tendency for urinary levels among these applicator bystanders was from less than LOD to 3 ppb.

**Table 2:** Bystanders and rural populations, source of exposure expected to be indirect, in µg/L (ppb).

					Selected percentiles					
							
Source	Group	Number of subjects	LOD	% < LOD	25th	GM (AM)	50th	75th	Max	Comment
Alexander et al. (2007)	Crop applicator, pre-application	34	1.0	30	NR	4	2	NR	231	
Alexander et al. (2007)	Children of applicator day 2	52	1.0	11	NR	4	3	NR	263	20 children were present during the application process, 4 had opportunity for direct contact.
Alexander et al. (2007)	Spouses of applicator day 2	34	1.0	32	NR	1	1	NR	25	8 spouses were present during the application process, 1 had opportunity for direct contact.
Arbuckle et al. (2002)	Crop applicator did not use 2,4-D	83	1.0	NR	<LOD	1	<LOD	1	66	
Arbuckle et al. (2002)	Children of applicator day 2	92	1.0	86	NR	(1.9)	NR	<LOD	100	Child with highest concentration was directly involved in the handling of the herbicide.
Arbuckle and Ritter (2005)	Spouses of applicator day 2	125	1.0	86	NR	1(2)	<LOD	<LOD	100	Spouse with the highest concentration helped with the handling of the herbicide
Arcury et al. (2007)	Children of farm worker	60	0.2	58	NR	NR	<1	NR	NR	Only % detected reported
Arcury et al. (2010)	Farm worker	196 (784 samples)	0.2	2	NR	NR	NR	NR	NR	Only % detected reported, 4 samples per person, 30% had all 4 detections
Bakke et al. (2009)	Crop applicator of atrazine, Pre season	30 (61 samples)	0.2	16	<LOD	1(3)	NR	NR	NR	90th percentile = 4
Bakke et al. (2009)	Crop applicator of atrazine, planting season	30 (157 samples)	0.2	4	NR	3(23)	NR	NR	NR	90th percentile = 67
Bakke et al. (2009)	Extension agent, all seasons	10 (49 samples)	0.2	53	<LOD	<1(<1)	NR	NR	NR	90th percentile = 2
Cooper et al. (2001)	Farm worker at pregnancy delivery	9	20	89	NR	NR	NR	NR	120	
Curwin et al. (2005)	Crop applicator, did not use 2,4-D	27	0.2	30	<LOD	<1	NR	NR	NR	
Curwin et al. (2005)	Farmer, 2,4-D sprayed by others	4	0.2	0	NR	2	NR	NR	NR	
Curwin et al. (2005)	Nonfarmer	45	0.2	31	<LOD	<1	NR	NR	NR	
Garry et al. (2001)	Licensed applicator, did not apply	15	0.6	80	<LOD	(0.5)	<LOD	<LOD	2	
Harris et al. (1992)	Household members, turf application, did not apply 2,4-D	19	NR	100	<LOD	<LOD	<LOD	<LOD	<LOD	A controlled study
Panuwet et al. (2008)	Crop applicator (Pon Yaeng) did not use 2,4-D	67	0.2	91	<LOD	NC	<LOD	<LOD	30	
Panuwet et al. (2008)	Crop applicator, (Inthakhin), not timed with application	69	0.2	35	<LOD	<1	<1	3	598	

AM, arithmetic mean; GM, geometric mean; LOD, limit of detection; NC, not calculated; NR, not reported.

### Manufacturers and applicators, direct exposure

The occupationally exposed include individuals working in 2,4-D application on crops, forests and turf and 2,4-D manufacture (Table 3). This group is highly heterogeneous with respect to exposure due to their varying opportunity for direct exposure to 2,4-D. Most have detectable urinary levels but not all. The data for crop and forestry applicators tend to be skewed with geometric means between 5 to 45 ppb, but maximum levels from 410 to 2500 ppb (Knopp & Glass, 1991; Garry et al., 2001; Arbuckle et al., 2002; Curwin et al., 2005; Alexander et al., 2007; Panuwet et al., 2008; Bhatti et al., 2010; Tomas et al., 2010). The unprotected turf applicators of liquid 2,4-D had the highest urinary levels reported in a controlled study (Harris et al., 1992). The highest levels in another turf study were observed during the spring season, compared to the summer and fall (Harris et al., 2010). Other exposure studies of professional turf applicators reported internal dose estimates and are not directly comparable to the studies in Table 3 (Harris et al., 2002, 2005). However, modeling of the variation of exposure in these reports confirmed that doses were Influenced by type of spray nozzle and use of gloves whereas job title alone was a poor determinant. Only one older study evaluated exposure among manufacturers in the 1980s (Knopp, 1994). This population had the highest range with a maximum of 12,963 ppb.

**Table 3:** Applicators, source of exposure expected to be direct, in µg/L (ppb).

					Selected percentiles					
Source	Group	Number of subjects	LOD	% < LOD	25th	GM (AM)	50th	75th	Max	Comment
Alexander et al. (2007)	Crop applicator, Day 2	34	1	3	13	45	80	145	2236	Levels associated with glove use, repairing equipment and acres treated.
Arbuckle et al. (2002)	Crop applicator, Day 1	43	1	52	1	5	6	13	410	Levels associated with protective gear, application equipment, handling practice, and personal hygiene practice.
Bhatti et al. (2010) Figgs et al. (2000)	Seasonal applicator	31 (136 samples)	1	NR		63(259)	94		2857	16% of between worker variance explained by method, month, concentration, gloves
Curwin et al. (2005)	Crop applicator	16	0.2	6		13				Levels associated with time since application, amount applied, and acres treated
Garry et al. (2001)	Forestry applicator	24	0.6	1	20	49	61	145	1700	Levels associated with application method
Harris et al. (1992)	Turf home applicators; granular, unprotected	11	4	1/11	<LOD	(9.8)	<LOD	<LOD	108	
Harris et al. (1992)	Turf home applicators; granular, unprotected	9	4	1/9	<LOD	(19)	<LOD	<LOD	169	
Harris et al. (1992)	Turf home applicators; liquid, unprotected	11	4	2/11	<LOD	(9)	<LOD	<LOD	63	
Harris et al. (1992)	Turf home applicators; liquid, unprotected	9	4	6/9	85	(204)	111	289	744	
Harris et al. (2010)	Turf applicators 24-h urine samples	135 (513 samples)		42			15		3658	
Knopp (1994)	Manufacturers, 1985–1986,	41	1	0	332	(1345)	731	1391	12,963	Within frst two hours of working shift (Friday)
Knopp and Glass (1991)	Crop applicator, Day 3	2	0.5	0		(1317)			2480	
Knopp and Glass (1991)	Forestry applicator, Pilot, Day 1	1	0.5	0	NA	NA	NA	NA	52	
Knopp and Glass (1991)	Forestry, mixer-loader, Day 1	1	0.5	0	NA	NA	NA	NA	365	
Lerda and Rizzi (1991)	Crop applicator	32	NR	NR	NR	(9000)	NR	NR	NR	Poor information on methods
Panuwet et al. (2008)	Crop applicator, not timed with application	45		35	<LOD	<1	<1	3	598	
Tomas et al. (2010)	Crop applicator, Day 5	26	0.2	0	NR	17	NR	NR	2500	Lower GM levels associated with glove use and application method.

AM, arithmetic mean; GM, geometric mean; LOD, limit of detection.

### Summary of biomonitoring

In general, urinary levels of 2,4-D are correlated with individual behavior, performed tasks and opportunity for direct contact with the herbicide. Urinary 2,4-D levels are near or below 1 ppb in most of the sampled general populations. Many of the studies of bystanders and applicators reported information on activities, use of protective equipment and application methods. The highest urinary levels were observed in “bystanders” with opportunity for direct contact with 2,4-D by assisting with the application, being present during the application, handling the herbicide (Arbuckle et al., 2004, 2005; Alexander et al., 2007). Work practices among applicators were also demonstrated to predict urinary 2,4-D. These include glove use, repairing equipment, application method, acres treated and personal hygiene practices. In seasonal applicators, Bhatti et al. (2010) observed that these factors explained only 16% of the variance between workers suggesting that other factors remain to be identified.

Exposure classifications in epidemiology studies can be strengthened and validated by means of high quality biomonitoring data. Currently, pesticide exposure in epidemiology studies is frequently based upon unvalidated self-reported use and/or activities with probable contact with herbicides. From a given publication, it is often difficult to differentiate if a participant applied 2,4-D or was near an application and thus assigned as “exposed.” Epidemiologists are always concerned about reporting and recall bias. However, the 2,4-D urinary biomonitoring studies suggest that we can also be focused on collecting better data relevant to the application. Biomonitoring studies of applicators and their spouses have established that contact with 2,4-D through mixing, loading and applying is a strong determinant of internal exposure whereas living near the application is not (Arbuckle et al., 2002; Arbuckle & Ritter, 2005; Alexander et al., 2007).

For example, in their study of NHL, Hartge et al. (2005) measured levels of 2,4-D in household dust from vacuum cleaner bags and subsequently calculated risk estimates by dust levels in quintiles from below detection to greater than 10,000 ng/g. However, without corresponding urinary monitoring, no internal dose estimates are possible. Levels of 2,4-D in carpet dust and urine were collected in another study by Morgan et al. (2008). Although 2,4-D was detected in more than 80% of the samples, with a maximum level of 21,700 ng/g, urine levels were not statistically significantly correlated with the 2,4-D concentrations in the dust samples. Whereas, 2,4-D can be identified in household dust, detection has not been demonstrated to be a good predictor of exposure based on urinary levels, and its use in epidemiology studies should be viewed with caution. This is consistent with the conclusions of Curwin et al. (2005) that the key determinant for exposure is actually applying the pesticide of interest.

In conclusion, the biomonitoring data for 2,4-D provide important information about the plausibility and validity of exposure estimates in the epidemiology literature.

## Human health endpoints

### Cancer

#### Cancers afecting the lymphatic system (NHL, MM, leukemia)

The focus of cancer studies and 2,4-D has historically addressed the lymphatic system, and primarily non-Hodgkin lymphoma (NHL). The current decade is no different with 9 case–control studies (12 publications) reporting on lymphohematopoeitic cancers (Table 4). The largest NHL study is the Italian multicenter investigation with 1575 cases reported in 2003 and 1925 cases by 2006 (Miligi et al., 2003, 2006). The estimate among all participants demonstrated no increased risk from 2,4-D use (OR = 0.9, 95% CI = 0.5–1.8). The odds ratios were not significant when stratifed by men (0.7, 95% CI = 0.3–1.9) and women (1.5, 95% CI = 0.4–5.7). Nine cases and 3 controls reported 2,4-D use (greater than low) and not using protective equipment. The large odds ratio of 4.4 was statistically significant but imprecise (95% CI = 1.1–29.1).

**Table 4:** Carcinogenicity: summary of findings of 2,4-D exposure and cancer.

Author, year	Design	Exposure	Exposed cases	Results	Comments/conclusions
Alavanja et al. (2003)	Nested C–C Prostate cancer	Applied 2,4-D (self report)	NR	Data not shown	No signifcant increase, AHS Very large sample size (566 cases, 54 766 controls)
Band et al. (2011)	Nested C–C Prostate cancer	Ever exposed to 2,4-D (self report and job exposure matrix)	11	OR = 2.72 (1.12–6.57) Dose response not signifcant (not shown)	Results inconsistent. Large sample size (1153 cases and 3999 controls
Boers et al. (2010)	Cohort	Worked in chlorophenoxy		Hazard ratios for Factory A:	No signifcant increase
	All cancers	manufacturing	81	All cancers: 1.31 (0.86–2.01)	Moderate cohort (Factory A = 539 exposed, 18 811 person years and Factory B = 411 exposed, 12 946 person years)
	NHL		4	NHL: 0.92 (0.19–4.47) (1 case in Factory B)	Not specifc to 2,4-D, exposures include 2,4,5-T, MCPA, other pesticides and possibly TCDD.
	Stomach		5	Stomach: 2.23 sibly TCDD. (0.38–13.20)	
	Melanoma		3	Melanoma: 0.72 (0.07–7.03)	
	Prostate		6	Prostate: 2.93 (0.61–14.15)	
Burns et al. (2011)	Cohort	Worked in 2,4-D		SIR for Cohort 2	No signifcant increase
	All cancers	manufacturing	244	All cancers: 0.88 (0.78–1.00)	Signifcant defcit of prostate cancer
	NHL		14	NHL 1.36 (0.74–2.29) 14 cases	Other exposure include benzene, asbestos and possibly TCDD
	Stomach		4	Stomach: 0.85 (0.23–2.18)	Moderate cohort of 1256 workers, 23 354 person years)
	Melanoma		9	Melanoma: 1.06 (0.48–2.01)	
	Brain cancer		3	Brain: 0.87 (0.17–2.54)	
	Prostate		62	Prostate: 0.74 (0.57–0.94)	
Carreón et al. (2005)	C-C	Exposed to 2,4-D on a farm (self-report)	24	OR = 0.9 (0.5–1.6) including proxy	No signifcant increase
	Glioma		11	OR = 0.8 (0.4–1.6) excluding proxy	Moderate sample size (341 cases, 527 controls)
Chiu et al. (2006)^a^; See Lee et al. (2004a) and De Roos et al. (2003)	C–C NHL by t(14:18) status	Used phenoxy herbicides (self-report)		T(14:18) positive No use: OR = 5.0 (1.2–20.8)	Results inconsistent. Not specifc to 2,4-D
			14	Used: OR = 2.9 (1.0–8.4) T(14:18) negative No use: OR = 1.5 (0.5–4.8)	Risk from no use higher than risk of use Large sample size (172 cases, 1432 controls) All pesticide analyses higher for t(14:18) positive status than negative
			Used OR = 0.8 (0.4–1.6)		
De Roos et al. (2003)^a^; See Chiu et al. (2006) and Lee et al. (2004a)	C–C NHL	Used 2,4-D (self-report)	123	OR = 0.9 (0.6–1.2) No increase both malathion and 2,4-D	No signifcant increase Large sample size (870 cases, 2569 controls)
Dennis et al. (2010)	Nested C–C Melanoma	Applied 2,4-D (self report)	NR	data not shown	No signifcant increase, AHS Very large sample size (150 cases and 24 554 noncases)
Engel et al. (2005)	Nested C–C Breast cancer	Applied 2,4-D (self report)	41	Adj RR 0.8 (0.6–1.1) (also by state and menopausal status)	No signifcant increase, AHS Very large sample size (309 cases, 30 145 noncases)
Eriksson et al. (2008)	C-C	Used 2,4-D and/or 2,4,5-T (self-report)	33	Multivariate OR = 1.24 (0.68–2.26)	No signifcant increase
	NHL and specifc entities		21	OR = 2.08 (0.99–4.38) for ≤ 29 days	Large sample size (910 cases, 1016 controls)
			12	OR = 1.33 (0.57–3.13) for >29 days. Non signifcant Ors for 8 entities	Signifcant results for phenoxy herbicides driven by MCPA results.
Flower et al. (2004)	Nested C–C	Parent used 2,4-D (self-report by parent)	7	Mother used OR = 0.72 (0.32–1.60)	No signifcant increase, AHS
	Cancer in children		26	Father used OR = 1.29 (0.71-2.35)	Large sample size (17 280 children) 50 cancer cases, 2 NHL cases
Hartge et al. (2005)	C-C	2,4-D in carpet dust	257	OR = 1.10 (0.78–1.55) <500 ng/g in dust	No signifcant increase
	NHL		86	OR = 0.91 (0.58-1.45) 500-999 ng/g	Large sample size (1321 cases, 1057 controls; with dust 679 cases, 510 controls)
			165	OR = 0.66 (0.45-0.98) 1000-9999 ng/g	
			24	OR = 0.82 (0.41-1.66) >10,000 ng/g	
Hohenadel et al. (2011); See McDuffie 2001, 2005a	C-C	Used 2,4-D (self-report)	49	2,4-D alone OR = 0.94 (0.67-1.33)	Risk only in combination
	NHL		61	Malathion and 2,4-D OR = 2.06 (1.45–2.93)	Large sample size (513 cases, 1506 controls)
Lee et al. (2004a)^a^; See Chiu 2006 and	C-C	Used 2,4-D (self-report)	172	Non asthmatics OR = 1.0 (0.8-1.3)	No signifcant increase
De Roos et al. (2003)	NHL		17	Asthmatics OR = 1.3 (0.7–2.5)	Large sample size (872 cases, 2381 controls) Asthmatics tended to have higher Ors for many other pesticides
Lee et al. (2005)	C-C	Used 2,4-D (self report)	25	Proxy OR = 3.3 (1.5–7.2)	Potential recall bias in proxies
	Glioma		7	Self OR = 0.6 (0.2–1.6)	Moderate sample size (251 cases, 498 controls)
Lee et al. (2004b)	C-C	Used 2,4-D (self-report)	27	Stomach OR = 0.8 (0.4–1.3)	No signifcant increase
	Stomach, Oesophagus		20	Oesophagus OR = 0.7 (0.4–1.2) Proxy respondents for 80% of stomach and 76% of oesophageal cases	Moderate sample size (170 stomach, 137 oesophagus, 502 controls) Potential recall bias in proxies
Lee et al. (2007)	Nested	Used 2,4-D (self-report)	135	Colon OR = 0.6 (0.4–0.8), also signifcant by lifetime exposure days (*p* = 0.011)	Signifcant inverse association for colon, AHS
	C-C Colorectal		69	Rectum OR = 1.1 (0.6–2.0)	Very large sample size (305 cases, 56 508 noncases)
McDufe et al. (2001)^a^	C-C	Used 2,4-D (self report)	111	Adj OR = 1.32 (1.01-1.73)	Results inconsistent
	NHL	More than 10 hours per year		No dose response by days per year Conditional, most parsimonious model excluded phenoxy herbicides.	Large sample size (517 cases, 1506 controls) Results confounded by other factors
McDufe et al. (2005)^a^	C-C NHL	Used 2,4-D (self-report) More than 10 hours per year	37 73 20	Exp to 2,4-D, no DEET OR = 1.05 (0.69-1.6) Both 2,4-D and DEET OR = 1.17 (0.84–1.64) Glove,2,4-D and DEET OR = 1.77 (0.90–3.45)	No signifcant increase Large sample size (513 cases, 1506 controls) OR highest when used rubber gloves.
Miligi et al. (2003)^a^	C-C	Exposed to 2,4-D (Expert assessment from self-report)	6	Men OR = 0.7 (0.3–1.9)	No signifcant increase
	NHL		7	Women OR = 1.5 (0.4–5.7)	Large sample size (1575 cases, 1232 controls)
Miligi et al. (2006); See Miligi 2003^a^	C-C	Exposed to 2,4-D (Expert assessment from self-report)	17	Overall OR = 0.9 (0.5-1.8)	Results imprecise
	NHL		9	Lack of protective equipment OR = 4.4 (1.1–29.1)	Large sample size (1925 cases, 1232 controls)
Mills and Yang (2005)	C-C	Work records matched with use registry		Cases diagnosed <1995	Results inconsistent by diagnosis date. OR's similar for low and high use.
	Breast cancer		12	OR = 0.61 (0.20–1.86) for low use	Exposure subject to misclassifcation: assumed from working in felds but metrics not timed with application and entry.
			8	OR = 0.62 (0.23-1.69) for high use Cases diagnosed ≥1995	Moderate sample size (128 cases, 640 controls)
			19	OR = 2.16 (0.95–4.93) for low use	
			21	OR = 2.14 (1.06–4.32) for high use	
Mills et al. (2005)	C-C	Work records matched with use registry	NR	NHL High (v Low) OR = 3.8 (1.85-7.81)	Results imprecise (only 15% exposed to 2,4-D)
	NHL, MM, Leukemia			NHL extranodal OR = 9.73 (2.68–35.3)	Exposure subject to misclassifcation: assumed from working in felds but metrics not timed with application and entry.
				NHL males OR = 3.79 (1.58–9.11) NHL females OR = 5.23 (1.3–20.9) *N* = 15 Leukemia OR = 1.03 (0.41–2.61) MM, “no elevated risks”	Moderate sample size (60 NHL, 51 leukemia, 20 MM cases and 5 controls per case)
Mills and Yang (2007)	C-C	Work records matched with use registry	17	Signifcant at lowest pounds of use	Results inconsistent for reference and no dose response.
	Gastric (stomach) cancer		14	OR = 2.16 (1.02-4.56) for 1–14 lbs	Moderate sample size (100 cases, 210 controls)
			11	OR = 1.57 (0.71–3.51) for 15–86 lbs OR = 2.09 (0.87–5.05) for 86–1950 lbs No increase using low as the reference.	Exposure subject to misclassifcation: assumed from working in felds but metrics not timed with application and entry.
Pahwa et al. (2006) and Pahwa et al. (2011)	C-C	Used 2,4-D (self report)	57	HL OR = 0.96 (0.67–1.37)	No signifcant increase
	HL, MM, STS	More than 10 hours per year	80	MM OR = 1.21 (0.89–1.65)	Large sample size (316 HL, 342 MM, 357 STS, 1506 controls)
			69	STS OR = 0.97 (0.71–1.32) No increases if both DEET and 2,4-D	
Schroeder et al. (2001)	C-C	Used phenoxy herbicides (self report)	17	T (14:18) positive OR = 0.9 (0.5–1.5)	No signifcant increase
	NHL specifc entities		30	T (14:18) negative OR = 1.1 (0.7–1.5)	Not specifc to 2,4-D Large sample size (622 cases, 182 cases with translocation status, 1245 controls)

a Same population.

C–C, case–control study; HL, Hodgkin lymphoma; MM, Multiple myeloma; NR, not reported; STS, soft tissue sarcoma.

Case–control size: Very large (>10 000); Large (>1000); Moderate (>100). Cohorts small (<10 000 person years at risk).

Another large NHL case–control study from Sweden, (Eriksson et al., 2008) reported no statistically significant increased risk for 2,4-D and/or 2,4,5-T use among 910 cases and 1016 controls. Tere was no increase in odds ratios by days used nor any significant increase by any of eight cancer entities. A statistically significant risk for NHL and phenoxy herbicides, in general, was Influenced by the significant odds ratios for MCPA.

Data were pooled from three previously published case–control studies in Kansas (Hoar et al., 1986), Nebraska (Zahm et al., 1990) and Iowa and Minnesota (Cantor et al., 1992) resulting in 870 NHL cases and 2569 controls (De Roos et al., 2003; Lee et al., 2004a). There were 123 cases and 314 controls who reported using 2,4-D. No significant increases for using 2,4-D and NHL were reported in analyses to address possible associations among asthmatics and adjusting for co-exposure to other pesticides (De Roos et al., 2003; Lee et al., 2004a). Another similarly sized study (679 cases and 510 controls) reported no significant increase in risk of NHL and 2,4-D in carpet dust (Hartge et al., 2005).

A large case–control study of 517 NHL cases and 1506 controls from six Canada provinces observed a statistically significant odds ratio for 2,4-D use after adjusting for medical variables (OR = 1.32, 95% CI = 1.01–1.73) (McDufe et al., 2001). However, multivariate analyses and stratification by days of use yielded no statistically significant results. In their analyses of the use of the insect repellent, DEET (N,N-diethyl-m-toluamide) the authors reported no statistically significant increase for NHL and 2,4-D (McDufe et al., 2005). The odds ratio for using gloves, 2,4-D and DEET was 1.77 (95% CI = 0.90–3.45). The authors discuss that DEET may increase permeability of rubber gloves. However, one would expect a similar or equal risk compared to no glove use, not higher. The analyses of these data by Hohenadel et al. (2011) reported an increased odds ratio among respondents who used both malathion and 2,4-D (OR = 2.06, 95% CI = 1.45–2.93). This is in contrast with the pooled study of De Roos et al. (2003) that reported no association for use of both pesticides.

The studies by Schroeder et al. (2001) and Chiu et al. (2006) evaluated t(14:18) subtypes of NHL in case–control studies in Iowa/Minnesota, and Nebraska. Neither evaluated 2,4-D specifically and only reported use of phenoxy herbicides. Schroeder et al. (2001) reported no increased risk for the t(14:18) positive subtype (OR = 0.9, 95% CI = 0.5–1.5).

The smallest case–control study published in the past decade was the United Farm Worker analysis of 60 cases of NHL (Mills et al., 2005). With the small sample size and only 15% of the subjects classified as exposed to 2,4-D, the results are imprecise. For example, the confidence interval for the NHL-extranodal association (OR = 9.73, 95% CI = 2.68–35.3) is very wide (confidence interval ratio = 13).

Two occupational cohort studies of workers in 2,4-D and phenoxy herbicide manufacturing have been updated since the 2001 review. Neither study reported a statistically significant increase in NHL (Boers et al., 2010; Burns et al., 2011). The cohort of foresters was too small for a meaningful evaluation of 3 NHL cases (Törn et al., 2000).

Other lymphohematopoetic cancers such as leukemia, Hodgkin lymphoma and multiple myeloma were also investigated and also were not found to be associated with 2,4-D exposure (Mills et al., 2005; Pahwa et al., 2006; Boers et al., 2010; Burns et al., 2011).

In summary, since 2001, there have been 9 epidemiology studies to investigate the association of 2,4-D and NHL and only 2 reported a statistically significant positive association (McDufe et al., 2005; Mills et al., 2005). The largest, most robust studies found no dose response and no statistically significant increase in use of 2,4-D and NHL. The few statistically significant results reported were internally inconsistent with other dose and multi-variate analyses in the same study, were imprecise and were not confirmed in studies of other populations. These studies provide inconsistent evidence of increased risk of NHL or other cancers of the lymphatic system.

#### Other cancers

The inconsistencies in the epidemiology data are often attributed to poor quality and inadequate sample size. The Agricultural Health Study (AHS) was designed to address these inadequacies by enrolling approximately 90 000 farmer applicators and their spouses. Tere are more than 100 publications from the AHS (http://aghealth.nci.nih.gov/). Although the AHS has not reported on 2,4-D applicators as a group specifically, nested case–control studies of prostate cancer, breast cancer, colorectal cancer, melanoma of the skin and childhood cancer have evaluated 2,4-D. None of these studies reported an association with 2,4-D. Each is detailed below by cancer site. In addition to the AHS publications, other studies that report these cancer sites and 2,4-D are also discussed (Table 4).

The prostate cancer case–control study nested in the AHS reported no significant increase use of 2,4-D by cases (Alavanja et al., 2003). The much smaller Dutch manufacturing cohort also reported no significant increase (Boers et al., 2010) and the other manufacturing cohort reported a statistically significant defcit of prostate cancer among 2,4-D workers (Burns et al., 2011). Exploratory analyses in a recent case–control study were strong and statistically significant (OR = 2.72, 95% CI = 1.12–6.57) but with only 12 exposed cases, dose-response analyses were not examined (Band et al., 2011). Together, these studies provide inconsistent evidence of increased risk of prostate cancer and 2,4-D exposure.

No significant increase of breast cancer was reported by the Engel et al. (2005) analysis of the AHS women who reported using 2,4-D. This is in contrast to a UFW case–control study that reported a statistically significant odds ratio of 2.14 (95% CI = 1.06–4.32) for cases diagnosed after 1994 (Mills, 2005). However, the odds ratio was inexplicably less than expected for early diagnosed (before 1994) cases (OR = 0.6, 95% CI = 0.23–1.69). There was no dose response since the ORs were similar for low and high use. Exposure misclassification may exist in this study since exposures were assumed for all farm workers and the determination of 2,4-D exposure was not timed by application or entry into the feld. These studies of breast cancer in women are inconsistent.

Cancers of the colon and rectum were evaluated in the AHS by Lee et al. (2007). No significant increase was reported for rectal cancer and a statistically significant inverse dose response by lifetime exposure days (*p* = 0.011) was observed for colon cancer and 2,4-D. The manufacturing cohorts of Boers et al. (2010) and Burns et al. (2011) reported no significant increases. These studies provide no evidence of increased risk of colorectal cancer.

Dennis et al. (2010) reported no significant increase in or risk melanoma of the skin associated with 2,4-D exposure. This is consistent with both the Dutch and US 2,4-D manufacturing cohorts (Boers et al., 2010; Burns et al., 2011). These studies provide no evidence of increased risk of melanoma.

A study of children of participants of the AHS who ever applied 2,4-D was reported by Flower et al.(2004). No sig-nifcant increase in cancer was observed.

The association of 2,4-D exposure and two other cancer sites have been addressed by other non AHS studies. A statistically significant association was reported by Lee et al. among glioma proxy respondents (OR = 3.3, 95% CI = 1.5–7.2) but not among self respondents (OR = 0.6, 95% CI = 0.2–1.6). There may be appreciable recall bias (Lee et al., 2005). No significant increase for 2,4-D and glioma was reported by Carreón et al. (2005). The risk of brain cancer was also not increased in the 2,4-D occupational cohort (Burns et al., 2011). These studies provide inconsistent evidence of increased risk of brain cancer.

No significant association between stomach and oesophageal cancers and 2,4-D exposure was reported in a case–control study (Lee et al., 2004b) and the results were inconsistent for stomach cancer in the UFW case–control study by Mills et al. (2007). Neither the Dutch nor the US occupational cohort study reported significant increased risk of stomach cancer (Boers et al., 2010; Burns et al., 2011). These studies provide inconsistent evidence of increased risk of stomach cancer

### Reproductive toxicity

Using a cross sectional design, Swan et al. (2003) evaluated urinary 2,4-D levels, semen quality, sperm concentration, morphology, and motility in a multi-center study of partners of pregnant women (Table 5). They found no significant associations in semen quality, concentration, morphology or motility with 2,4-D levels. An exposure study of 2,4-D farmers did identify 2,4-D in the sperm of study participants (Arbuckle et al., 1999). These findings are difficult to interpret with respect to farmer practices because detectable levels were found in over 50% of the subjects with no reported use of 2,4-D and nearly half of those who did report using 2,4-D were below detection (summarized in Table 5).

**Table 5:** Reproductive toxicity: summary of findings of 2,4-D exposure and reproductive endpoints.

Author	Design	Exposure	Exposed cases	Results	Comment/conclusions
Arbuckle et al. (2001)^a^; Arbuckle et al. (1999)^a^	Nested C–C Spontaneous abortion (SAB)	Used 2,4-D in farming during pregnancy period	26	<12 weeks gestation Preconception exposure OR = 1.3 (0.9–2.0)	Results inconsistent Large sample size (395 SAB among 3936 pregnancies)
			9	Post conception exposure OR = 0.6 (0.3–1.2)	Related analysis of Arbuckle 1999, “results were sensitive to the cutpoint used” (12 vs. 13 weeks gestation)
			22v5	Pre vs. Post OR = 2.9 (1.1–8.0)	
			11v14	12–19 weeks, Pre vs. Post OR = 0.5 (0.2–1.1)	
Swan et al. (2003)	Nested C–C	Urinary 2,4-D (≥0.1 µg/g cr)	5	Low semen quality OR = 0.8 (0.2–3.0) (Missouri, no exposed cases in Minnesota)	No significant increase
		Semen quality		Concentration, morphology, % motile (all *p* values >0.05)	Small sample size (65 cases and 101 controls with semen samples)
Waller et al. (2010)	C–C	2,4-D in surface water (>70 µg/L)	NR	OR = 1.05 (0.81–1.37) <10 km to water	No significant increase
		Gastroschisis (birth deffect)			Large sample size (805 cases, 3616 controls)
Weselak et al. (2007)^a^	Nested C–C	Used 2,4-D in farming during pregnancy period		In ofspring:	Significant only for hayfever/allergies
	Allergies, hayfever in ofspring		21	Bronchitis OR = 1.46 (0.80–2.66)	Large sample size (805 children)
			24	Asthma OR = 0.74 (0.38–1.43)	Since a farming family, exposure not restricted to in utero.
			52	Hayfever/allergies OR = 1.66 (1.11–2.49)	
Weselak et al. (2008)^a^	Nested C–C	Used 2,4-D in farming during pregnancy period	7	Any deffect OR = 0.97 (0.42–2.25)	No significant increase
	Birth deffects		6	Pre-conception OR = 0.60 (0.25–1.46)	Large sample size (118 malformation in 3412 pregnancies

a Same cohort.

C–C, case–control study; NR, not reported.

To our knowledge, the only other study to evaluate semen quality and 2,4-D exposure is a small study of 32 farmers and 25 controls (Lerda & Rizzi, 1991). Using urine and sperm samples collected during a growing season, the authors reported differences in sperm quality at one stage but not another among 2,4-D exposed farmers compared to controls. The validity and generalizability of this study, however, are questionable. The study conduct was poorly described making evaluation of the study quality impossible. No information was provided about timing of either the urine or semen collection with respect to 2,4-D application. Lastly, the mean urinary levels of the exposure group at 9.0 mg/L (9000 ppb) are 200 to 500 times higher than the mean levels measured in other farmer studies (Alexander et al., 2007; Tomas et al., 2010). It is unclear if this is due to unique practices or incorrect analytical methods. Regardless, from a dose interpretation, these urinary levels are unanticipated for current farmers. These few publications provide no convincing evidence for sperm cell toxicity in humans from 2,4-D exposure.

Only one study evaluated rates of spontaneous abortion following use of 2,4-D during the pregnancy period (Arbuckle et al., 1999; Arbuckle et al., 2001). The overall results showed no association with 2,4-D use in the preconception period (OR = 1.2, 95% CI = 0.8–1.6) or the postconception period (OR = 1.0, 95% CI = 0.7–1.6). A single analysis comparing pre- and post-conception exposure less than 12 weeks gestation was statistically significant (OR = 2.9, 95% CI = 1.1–8.0). The results were sensitive to the use of 12 or 13 weeks as the cutpoint for exposure period suggesting a random finding.

Three studies evaluated birth deffects but none identified a significant increase with 2,4-D exposure (Schreinemachers, 2003; Weselak et al., 2008; Waller et al., 2010). A publication on respiratory endpoints in children whose parents used 2,4-D during the pregnancy period reported a significant prevalence of hay fever and/or allergies (OR = 1.66, 95% CI = 1.11–2.49). However, the study did not evaluate and cannot exclude exposure to allergens after birth (Weselak et al., 2007). These studies provide no evidence of increased risk of birth deffects due to 2,4-D exposure.

### Genotoxicity, including hormones and immune system

A few investigators have reported on cytogenetic damage among agricultural workers, but none was specific to 2,4-D exposure (Garaj-Vrhovac & Zeljezic, 2000, 2001, 2002; Gómez-Arroyo et al., 2000; Martínez-Valenzuela et al., 2009; Remor et al., 2009; Zeljezic & Garaj-Vrhovac, 2002). A small study of farmer applicators reported no association with micronuclei scores, lymphocyte phe-notypes and blood counts (Figgs et al., 2000). Some estimates of replicative index were significantly associated with urinary 2,4-D levels but others were not. These findings do not support the hypothesis of changes in immunological variables after a 2,4-D application (Faustini et al., 1996). Another small study identified an association with luteinizing hormone (LH) levels and urinary 2,4-D levels, but found no additional associations with hormone levels or chromosomal aberrations (Garry et al., 2001). Neither study finding has been confirmed by other independent investigations. No significant increase in antinuclear antibodies positivity among 2,4-D users was reported in the cross sectional study by Semchuk et al. (2007) (summarized in Table 6).

**Table 6:** Genotoxicity: summary of findings of 2,4-D exposure and genetic, hormone, and immune related endpoints.

Author	Design	Exposure	Exposed cases	Results	Comments/conclusions
Figgs et al. (2000)	Cross sectional	Urinary 2,4-D in farmer applicators	12	Micronuclei scores, lymphocyte phenotypes, blood counts not significant.	Results inconsistent
	Micronuclei, replicative index (RI), lymphocyte phenotypes, complete blood counts			Replicative index (RI)	Only RI significant before/after and appl/control, no dose response
				Before/after (*p* = 0.016) Applicator/control (*p* = 0.046) Trend by urinary level (*p* = 0.15)	Small sample size (12 applicators, 9 controls)
Garry et al. (2001)	Cross sectional	Urinary 2,4-D and work records	21	LH correlated with urinary levels (*p* = 0.006)	Small sample size (24 applicators, 15 controls)
	Hormone levels, chromosomes aberrations			FSH, testosterone, chromo-some aberrations not cor-related with urinary levels V(D)J rearrangement frequency correlated with urinary levels by application method	Exposure according to application method
Semchuk et al. (2007)	Cross sectional Antinuclear antibodies (ANA)	Used 2,4-D (occupational)	50 48	OR = 0.70 (0.27–1.77) ANA + OR = 0.30 (0.11–0.86), men only	No significant increase Moderate sample size (*N* = 208)

^a^Same cohort.

### Neurotoxicity

Parkinsonism, Parkinson's Disease and use of 2,4-D have been evaluated in four independent studies and five publications (Kamel et al., 2007; Dhillon et al., 2008; Hancock et al., 2008; Tanner et al., 2009; Tanner et al., 2011). A multicenter case–control study (519 cases and 511 controls) by Tanner et al. (2009) reported a strong and statistically significant increased risk of Parkinsonism among 2,4-D users (OR = 2.59, 95% CI = 1.03–6.48). However further analyses by job and exposure duration did not support a dose response. No significant increase risk of 2,4-D use and Parkinson's Disease was identified in the other studies (summarized in Table 7).

**Table 7:** Neurotoxicity.

Author, year	Endpoint	Exposure	Exposed Cases	Results	Comments/conclusions
Dhillon et al. (2008)	C–C	Used 2,4-D	17	OR = 1.2 (0.6–2.8)	No significant increase
	Parkinson's disease				Moderate sample size (100 cases, 84 controls)
Kamel et al. (2007)^a^	Nested C–C	Used 2,4-D	47	OR = 0.9 (0.5–1.8) prevalent	No significant increase, AHS
	Parkinson's disease		49	OR = 1.0 (0.5–2.1) incident	Very large sample size (83 prevalent cases, 78 incident cases, 79 557 controls)
Hancock et al. (2008)	C–C	Used chorphenoxy acid/ester	15	OR = 2.07 (0.69–6.23)	No significant increase
	Parkinson's disease				Large sample size (319 cases, 296 controls)
Tanner et al. (2009)	Parkinsonism	Used 2,4-D	16	OR = 2.59 (1.03–6. 48) No association by duration of use	Results inconsistent Moderate sample size (519 cases, 511 controls)
Tanner et al. (2011)^a^; See Kamel et al. (2007)	Nested C–C	Used 2,4-D	76	OR = 1.2 (0.57–2.4)	No significant increase, AHS
	Parkinson's disease				Moderate sample size (110 cases, 358 controls)

a Same cohort.

C–C, case–control study.

### General toxicity

Various respiratory endpoints have been addressed in certain groups within the AHS. These endpoints are all self-reported and include wheeze, farmer's lung, chronic bronchitis, asthma, and rhinitis. Only atopic asthma was associated with 2,4-D use in the AHS women and rhinitis (runny nose) in the commercial applicators. In the absence of a dose response and in the context of many co-exposures the implication of these associations for 2,4-D *per se* is unclear. No other publication has confirmed these associations (summarized in Table 8).

**Table 8:** General toxicity.

Author	Endpoint	Exposure	Exposed cases	Results	Comments/Conclusions
Hoppin et al. (2006a)^a^	Nested C–C Wheeze	Used 2,4-D in private farming and commercial application	NR	Farmers OR = 0.97 (0.86–1.10) Commercial OR = 0.99 (0.73–1.34)	No signifcant increase, AHS Very large sample size (17 920 farmers and 2255 commercial applicators, 19 and 22% with wheeze)
Hoppin et al. (2002)^a^	Nested C–C	Used 2,4-D in farming	2395	OR = 0.99 (0.88-1.11)	No signifcant increase, AHS
	Wheeze		77	OR = 1.25 (0.83–1.90) at highest use (>40 days)	Very large sample size (3838 cases, 16 630 controls) Highest OR at highest dose
Hoppin et al. (2006b)^a^	Nested C–C	Used 2,4-D in commercial application	225	Current use OR = 1.27 (0.96-1.68)	No signifcant increase, AHS Large sample size (486 cases, 1769 noncases)
Hoppin et al. (2007a)^a^	Nested C–C Farmer's lung	Used phenoxy herbicide	363	OR = 1.12 (0.77-1.64)	No signifcant increase, AHS Results not specifc to 2,4-D Large sample size (395 cases, 16 563 controls)
Hoppin et al. (2007b)^a^	Nested C–C Chronic bronchitis	Used 2,4-D in farming	78	OR = 1.10 (0.90-1.35)	No signifcant increase, AHS Very large sample size (654 cases, 20 254 noncases)
Hoppin et al. (2008)^a^	Nested C–C	Used 2,4-D in farming, women only	52	Atopic asthma OR = 1.53 (1.12–2.10)	Signifcant increase, AHS
		Asthma	66	Nonatopic OR 1.07 (0.82–1.41) Atopy alone OR = 1.20 (1.08–1.34)	Very large sample size (282 atopic asthma, 420 nonatopic, 3086 atopy, 25,112 controls)
			477	Growing up on farm OR = 0.55 (0.43–0.70)	OR > 1 for most pesticides reported
Schreinemachers (2010)	Cross sectional HDL, triglycerides, non-HDL, insulin, C-peptide, plasma glucose, thyroid stimulating hormone	Urinary levels in US population (> LOD)	102	Decreasing HDL (*p*< 0.05), increasing TSH (*p* < 0.05) among T4 < 8.5.	Only 14% above LOD
				Increasing triglycerides (*p* < 0.01), insulin (*p* < 0.05), c-peptide (*p* < 0.01) among HbA1c >5.1%	Moderate sample size (*N* = 727) Endpoints within normal range Cross sectional study with very low exposure.
Slager et al. (2009)^a^	Nested C–C Rhinitis (stufy, itchy, or runny nose in past 12 months)	Used 2,4-D as a commercial applicator	750 11	OR = 1.34 (1.09-1.64) OR = 0.69 (0.25-1.89) at highest dose (≥ 60 days)	Results inconsistent, no dose response, AHS Large sample size (1664 cases, months)
Valcin et al. (2007)^a^	Chronic bronchitis	Used 2,4-D in farming, women only	93	OR = 1.29 (1.02-1.63) adj for age, state OR = 1.20 (0.89–1.63) multivariate	Results attenuate with modeling, AHS Very large sample size (583 cases, 20 958 noncases)

a Same cohort.

C–C, case–control study; NR, not reported.

Another publication used the national data from the CDC to compare urinary levels in the US population to a battery of tests relevant to risk factors for heart disease. Whereas all the endpoints were within the normal range and only 14% of the subjects had 2,4-D levels above the LOD, some endpoints were statistically significantly associated with detectable urinary 2,4-D. As a cross sectional analysis of data collected at the same time for endpoints within normal, the etiologic implications are unclear. No other published study to date has tested these findings.

### Summary of epidemiology

**Figure 1:**
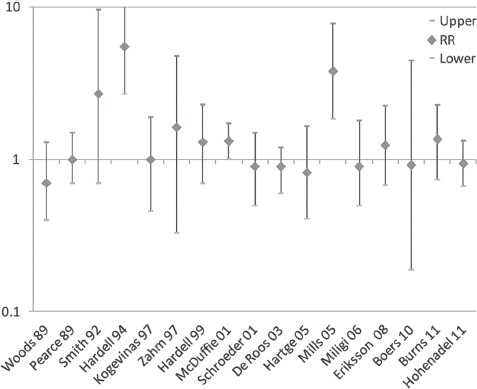
Relative risk estimates, lower and upper limits for epidemiology studies of NHL from 1989 to 2011. (See colour version of this figure online at www.informahealthcare.com/txc)

The 2,4-D epidemiology literature after 2001 related to carcinogenic reproductive, genotoxic, neurotoxic and general outcomes in humans is overwhelmingly negative. For cancer, the data are broad and consistent and give no credible or plausible evidence for any increased risk of cancer in humans associated with 2,4-D exposure. The most frequently reported cancer endpoint was NHL. The overall results for 7 studies reported prior to 2001 (Woods & Polissar, 1989; Pearce 1989; Smith & Christophers, 1992; Hardell et al., 1994; Kogevinas et al., 1997; Zahm 1997; Hardell & Eriksson 1999) and the 9 new studies reviewed since 2001 are shown in Figure 1. Unfortunately it was not possible to compare NHL results across studies by dose estimates. Several studies used duration as a proxy for increasing exposure but these maximum levels ranged from 7 days (McDufe et al., 2001), 29 days (Eriksson et al., 2008), 5 years (Burns et al., 2011), to 17 years (Chiu et al., 2006). Miligi et al. (2006) considered no use of protective equipment (i.e. gloves) to be the highest level whereas McDufe et al. (2005) categorized glove use with 2,4-D and DEET as the highest level. Overall, there are a few statistically significant positive observations, but the data are not consistent across studies, particularly in the past 10 years.

Statistical power to detect an association is Influenced by sample size of the study, numbers and magnitude of exposed and the strength of the association. One can play down a lack of statistical significance due to inadequate participants. Conversely, small studies with statistically significant and strong results may emphasize the role of statistical testing. A useful way to evaluate multiple results within and across studies is to compare the confidence interval ratio (CIR) (Poole, 2001). For example, as shown in Table 9, the female participants in the Italian study by Miligi et al. (2003 and 2006) might be assumed to have a higher risk of NHL than the men based upon the non significant odds ratios (OR = 1.5 vs. OR = 0.7). Further, the risk when not using protective equipment (OR = 4.4), attained statistical significance and was highlighted in the publication abstract. However, the estimates were imprecise as shown by their wide confidence intervals and large confidence interval ratios. The risk estimates may be subject to chance variation. The non significant estimate of 0.9 for the overall group is the most precise, and should be considered the most trustworthy with the least random error. Thus, precision is an important consideration along with replication and statistical significance when reviewing epidemiology study results.

**Table 9:** Comparison of results in an epidemiology study.

Group	OR	95% CI	Statistically significant	CI ratio
Men	0.7	0.3–1.9	No	6.3
Women	1.5	0.4-5.7	No	14.3
Overall	0.9	0.5-1.8	No	3.6–most precise
Lack of protective equipment	4.4	1.1-29.1	Yes	26.0–least stable

From Miligi et al. (2003, 2006).

In discussing specific health results, it is also important to take into account concepts related to reducing both random error and exposure misclassification. Investigators endeavor to maximize both sample size and exposure potential in selected populations. However, it is difficult to do one without sacrifcing the other. The very large AHS enrolled approximately 90 000 farmer applicators and their spouses but relied upon questionnaire based exposure determination. Urinary biomonitoring of a few AHS participants confirmed that internal doses of 2,4-D were well below exposure guidance values and that exposure was not uniform across applicators (Aylward et al., 2010; Tomas et al., 2010). Conversely, the occupational cohort studies of manufacturing workers of phenoxy herbicides have potentially higher and more frequent exposure but the sample sizes tend to be small. Much has been written about the problems and direction of exposure misclassification. In short, if unexposed participants are categorized as exposed, the risk estimates can be higher or lower (Tomas, 1995; Jurek et al., 2008). Keeping these strengths and weaknesses in mind, we looked for replication from one study to another with the view that no single epidemiology study can confirm or refute a putative association.

## Conclusion

Our interpretation of the epidemiology literature is that there is no convincing evidence for any chronic adverse effect of 2,4-D in humans. However, the epidemiology data are only a small portion of the information available. There is an abundance of information from the biomonitoring literature. These data inform us that persons with direct contact with 2,4-D have the highest exposure and that most non-agricultural populations have little to no measurable exposure. Guided by biomonitoring we can better control exposure and target populations that are the most exposed for future health research.

## References

[b1] Alavanja MC, Sandler DP, McDonnell CJ, Lynch CF, Pennybacker M, Zahm SH (1999). Characteristics of pesticide use in a pesticide applicator cohort: the Agricultural Health Study. Environ Res.

[b2] Alavanja MC, Samanic C, Dosemeci M, Lubin J, Tarone R, Lynch CF (2003). Use of agricultural pesticides and prostate cancer risk in the Agricultural Health Study cohort. Am J Epidemiol.

[b3] Alexander BH, Mandel JS, Baker BA, Burns CJ, Bartels MJ, Acquavella JF (2007). Biomonitoring of 2,4-dichlorophenoxyacetic acid exposure and dose in farm families. Environ Health Perspect.

[b4] Arbuckle TE, Schrader SM, Cole D, Hall JC, Bancej CM, Turner LA (1999). 2,4-Dichlorophenoxyacetic acid residues in semen of Ontario farmers. Reprod Toxicol.

[b5] Arbuckle TE, Savitz DA, Mery LS, Curtis KM (1999). Exposure to phenoxy herbicides and the risk of spontaneous abortion. Epidemiology.

[b6] Arbuckle TE, Lin Z, Mery LS (2001). An exploratory analysis of the effect of pesticide exposure on the risk of spontaneous abortion in an Ontario farm population. Environ Health Perspect.

[b7] Arbuckle TE, Burnett R, Cole D, Teschke K, Dosemeci M, Bancej CM (2002). Predictors of herbicide exposure in farm applicators. Int Arch Occup Environ Health.

[b8] Arbuckle TE, Cole DC, Ritter L, Ripley BD (2004). Farm children's exposure to herbicides: comparison of biomonitoring and questionnaire data. Epidemiology.

[b9] Arbuckle TE, Ritter L (2005). Phenoxyacetic acid herbicide exposure for women on Ontario farms. J Toxicol Environ Health Part A.

[b10] Arcury TA, Grzywacz JG, Barr DB, Tapia J, Chen H, Quandt SA (2007). Pesticide urinary metabolite levels of children in eastern North Carolina farmworker households. Environ Health Perspect.

[b11] Arcury TA, Grzywacz JG, Talton JW, Chen H, Vallejos QM, Galván L (2010). Repeated pesticide exposure among North Carolina migrant and seasonal farmworkers. Am J Ind Med.

[b12] Aylward LL, Morgan MK, Arbuckle TE, Barr DB, Burns CJ, Alexander BH (2010). Biomonitoring data for 2,4-dichlorophenoxyacetic acid in the United States and Canada: interpretation in a public health risk assessment context using Biomonitoring Equivalents. Environ Health Perspect.

[b13] Bakke B, De Roos AJ, Barr DB, Stewart PA, Blair A, Freeman LB (2009). Exposure to atrazine and selected non-persistent pesticides among corn farmers during a growing season. J Expo Sci Environ Epidemiol.

[b14] Band PR, Abanto Z, Bert J, Lang B, Fang R, Gallagher RP (2011). Prostate cancer risk and exposure to pesticides in British Columbia farmers. Prostate.

[b15] Bhatti P, Blair A, Bell EM, Rothman N, Lan Q, Barr DB (2010). Predictors of 2,4-dichlorophenoxyacetic acid exposure among herbicide applicators. J Expo Sci Environ Epidemiol.

[b16] Boers D, Portengen L, Bueno-de-Mesquita HB, Heederik D, Vermeulen R (2010). Causespecific mortality of Dutch chlorophenoxy herbicide manufacturing workers. Occup Environ Med.

[b17] Burns C, Bodner K, Swaen G, Collins J, Beard K, Lee M (2011). Cancer incidence of 2,4-D production workers. Int J Environ Res Public Health.

[b18] Cantor KP, Blair A, Everett G, Gibson R, Burmeister LF, Brown LM (1992). Pesticides and other agricultural risk factors for non-Hodgkin's lymphoma among men in Iowa and Minnesota. Cancer Res.

[b19] Carreón T, Butler MA, Ruder AM, Waters MA, Davis-King KE, Calvert GM (2005). Brain Cancer Collaborative Study Group. *Gliomas and farm pesticide exposure in women: the Upper Midwest Health Study*. Environ Health Perspect.

[b20] Centers for Disease Control and Prevention (CDC) (2009). http://www.cdc.gov/exposurereport/data_tables/index.html.

[b21] Chiu BC, Dave BJ, Blair A, Gapstur SM, Zahm SH, Weisenburger DD (2006). Agricultural pesticide use and risk of t(14;18)-defined subtypes of non-Hodgkin lymphoma. Blood.

[b22] Cooper SP, Burau K, Sweeney A, Robison T, Smith MA, Symanski E (2001). Prenatal exposure to pesticides: a feasibility study among migrant and seasonal farmworkers. Am J Ind Med.

[b23] Curwin BD, Hein MJ, Sanderson WT, Barr DB, Heederik D, Reynolds SJ (2005). Urinary and hand wipe pesticide levels among farmers and nonfarmers in Iowa. J Expo Anal Environ Epidemiol.

[b24] De Roos AJ, Zahm SH, Cantor KP, Weisenburger DD, Holmes FF, Burmeister LF (2003). Integrative assessment of multiple pesticides as risk factors for non-Hodgkin's lymphoma among men. Occup Environ Med.

[b25] Dennis LK, Lynch CF, Sandler DP, Alavanja MC (2010). Pesticide use and cutaneous melanoma in pesticide applicators in the agricultural heath study. Environ Health Perspect.

[b26] Dhillon AS, Tarbutton GL, Levin JL, Plotkin GM, Lowry LK, Nalbone JT (2008). Pesticide/environmental exposures and Parkinson's disease in East Texas. J Agromedicine.

[b27] Engel LS, Hill DA, Hoppin JA, Lubin JH, Lynch CF, Pierce J (2005). Pesticide use and breast cancer risk among farmers' wives in the agricultural health study. Am J Epidemiol.

[b28] Eriksson M, Hardell L, Carlberg M, Akerman M (2008). Pesticide exposure as risk factor for non-Hodgkin lymphoma including histopathological subgroup analysis. Int J Cancer.

[b29] Faustini A, Settimi L, Pacifci R, Fano V, Zuccaro P, Forastiere F (1996). Immunological changes among farmers exposed to phenoxy herbicides: preliminary observations. Occup Environ Med.

[b30] Figgs LW, Holland NT, Rothmann N, Zahm SH, Tarone RE, Hill R (2000). Increased lymphocyte replicative index following 2,4-dichlorophenoxyacetic acid herbicide exposure. Cancer Causes Control.

[b31] Flower KB, Hoppin JA, Lynch CF, Blair A, Knott C, Shore DL (2004). Cancer risk and parental pesticide application in children of Agricultural Health Study participants. Environ Health Perspect.

[b32] Gandhi R, Wandji S, Snedeker SM (2000). Critical evaluation of cancer risk from 2,4-D. Rev Environ Contam Toxicol.

[b33] Garabrant DH, Philbert MA (2002). Review of 2,4-dichlorophenoxyacetic acid (2,4-D) epidemiology and toxicology. Crit Rev Toxicol.

[b34] Garaj-Vrhovac V, Zeljezic D (2000). Evaluation of DNA damage in workers occupationally exposed to pesticides using single-cell gel electrophoresis (SCGE) assay. *Pesticide genotoxicity revealed by comet assay*. Mutat Res.

[b35] Garaj-Vrhovac V, Zeljezic D (2001). Cytogenetic monitoring of croatian population occupationally exposed to a complex mixture of pesticides. Toxicology.

[b36] Garaj-Vrhovac V, Zeljezic D (2002). Assessment of genome damage in a population of Croatian workers employed in pesticide production by chromosomal aberration analysis, micronucleus assay and Comet assay. J Appl Toxicol.

[b37] Garry VF, Tarone RE, Kirsch IR, Abdallah JM, Lombardi DP, Long LK (2001). Biomarker correlations of urinary 2,4-D levels in foresters: genomic instability and endocrine disruption. Environ Health Perspect.

[b38] Gómez-Arroyo S, Díaz-Sánchez Y, Meneses-Pérez MA, Villalobos-Pietrini R, De León-Rodríguez J (2000). Cytogenetic biomonitoring in a Mexican foriculture worker group exposed to pesticides. Mutat Res.

[b39] Hancock DB, Martin ER, Mayhew GM, Stajich JM, Jewett R, Stacy MA (2008). Pesticide exposure and risk of Parkinson's disease: a family-based case-control study. BMC Neurol.

[b40] Hardell L, Eriksson M, Degerman A (1994). Exposure to phenoxyacetic acids, chlorophenols, or organic solvents in relation to histopathology, stage, and anatomical localization of non-Hodgkin's lymphoma. Cancer Res.

[b41] Hardell L, Eriksson M (1999). A case-control study of non-Hodgkin lymphoma and exposure to pesticides. Cancer.

[b42] Harris SA, Solomon KR, Stephenson GR (1992). Exposure of homeowners and bystanders to 2,4-dichlorophenoxyacetic acid (2,4-D). J Environ Sci Health B.

[b43] Harris SA, Sass-Kortsak AM, Corey PN, Purdham JT (2002). Development of models to predict dose of pesticides in professional turf applicators. J Expo Anal Environ Epidemiol.

[b44] Harris SA, Sass-Kortsak AM, Corey PN, Purdham JT (2005). Pesticide exposures in professional turf applicators, job titles, and tasks performed: implications of exposure measurement error for epidemiologic study design and interpretation of results. Am J Ind Med.

[b45] Harris SA, Villeneuve PJ, Crawley CD, Mays JE, Yeary RA, Hurto KA (2010). National study of exposure to pesticides among professional applicators: an investigation based on urinary biomarkers. J Agric Food Chem.

[b46] Hartge P, Colt JS, Severson RK, Cerhan JR, Cozen W, Camann D (2005). Residential herbicide use and risk of non-Hodgkin lymphoma. Cancer Epidemiol Biomarkers Prev.

[b47] Health Canada (2008). http://www.hc-sc.gc.ca/cps-spc/pubs/pest/_decisions/rvd2008-11/index-eng.php.

[b48] Health Canada (2010). http:/www.hc-sc.gc.ca/ewh-semt/pubs/contaminants/chms-ecms/index-eng.php.

[b49] Hill RH, Head SL, Baker S, Gregg M, Shealy DB, Bailey SL (1995). Pesticide residues in urine of adults living in the United States: reference range concentrations. Environ Res.

[b50] Hoar SK, Blair A, Holmes FF, Boysen CD, Robel RJ, Hoover R (1986). Agricultural herbicide use and risk of lymphoma and soft-tissue sarcoma. JAMA.

[b51] Hohenadel K, Harris SA, McLaughlin JR, Spinelli JJ, Pahwa P, Dosman JA (2011). Exposure to multiple pesticides and risk of non-Hodgkin lymphoma in men from six Canadian provinces. Int J Environ Res Public Health.

[b52] Hoppin JA, Umbach DM, London SJ, Alavanja MC, Sandler DP (2002). Chemical predictors of wheeze among farmer pesticide applicators in the Agricultural Health Study. Am J Respir Crit Care Med.

[b53] Hoppin JA, Umbach DM, London SJ, Lynch CF, Alavanja MC, Sandler DP (2006a). Pesticides and adult respiratory outcomes in the agricultural health study. Ann N Y Acad Sci.

[b54] Hoppin JA, Umbach DM, London SJ, Lynch CF, Alavanja MC, Sandler DP (2006b). Pesticides associated with wheeze among commercial pesticide applicators in the Agricultural Health Study. Am J Epidemiol.

[b55] Hoppin JA, Umbach DM, Kullman GJ, Henneberger PK, London SJ, Alavanja MC (2007a). Pesticides and other agricultural factors associated with self-reported farmer's lung among farm residents in the Agricultural Health Study. Occup Environ Med.

[b56] Hoppin JA, Valcin M, Henneberger PK, Kullman GJ, Umbach DM, London SJ (2007b). Pesticide use and chronic bronchitis among farmers in the Agricultural Health Study. Am J Ind Med.

[b57] Hoppin JA, Umbach DM, London SJ, Henneberger PK, Kullman GJ, Alavanja MC (2008). Pesticides and atopic and nonatopic asthma among farm women in the Agricultural Health Study. Am J Respir Crit Care Med.

[b58] IARC (1986). Occupational exposures to chlorophenoxy herbicides. IARC Monographs on the Evaluation of the Carcinogenic Risk of Chemicals to Humans.

[b59] Kamel F, Tanner C, Umbach D, Hoppin J, Alavanja M, Blair A (2007). Pesticide exposure and self-reported Parkinson's disease in the agricultural health study. Am J Epidemiol.

[b60] Knopp D, Glass S (1991). Biological monitoring of 2,4-dichlorophenoxyacetic acid-exposed workers in agriculture and forestry. Int Arch Occup Environ Health.

[b61] Knopp D (1994). Assessment of exposure to 2,4-dichlorophenoxyacetic acid in the chemical industry: results of a five year biological monitoring study. Occup Environ Med.

[b62] Kogevinas M, Becher H, Benn T, Bertazzi PA, Bofetta P, Bueno-de-Mesquita HB (1997). Cancer mortality in workers exposed to phenoxy herbicides, chlorophenols, and dioxins. *An expanded and updated international cohort study*. Am J Epidemiol.

[b63] Kolmodin-Hedman B, Erne K (1980). Estimation of occupational exposure to phenoxy acids (2,4-D and 2,4,5-T). Arch Toxicol Suppl.

[b64] Kolmodin-Hedman B, Höglund S, Akerblom M (1983). Studies on phenoxy acid herbicides *I. Field study. Occupational exposure to phenoxy acid herbicides (MCPA, dichlorprop, mecoprop and 2,4-D) in agriculture*. Arch Toxicol.

[b65] Lee WJ, Cantor KP, Berzofsky JA, Zahm SH, Blair A (2004). Non-Hodgkin's lymphoma among asthmatics exposed to pesticides. Int J Cancer.

[b66] Lee WJ, Lijinsky W, Heineman EF, Markin RS, Weisenburger DD, Ward MH (2004). Agricultural pesticide use and adenocarcinomas of the stomach and oesophagus. Occup Environ Med.

[b67] Lee WJ, Colt JS, Heineman EF, McComb R, Weisenburger DD, Lijinsky W (2005). Agricultural pesticide use and risk of glioma in Nebraska, United States. Occup Environ Med.

[b68] Lee WJ, Sandler DP, Blair A, Samanic C, Cross AJ, Alavanja MC (2007). Pesticide use and colorectal cancer risk in the Agricultural Health Study. Int J Cancer.

[b69] Lerda D, Rizzi R (1991). Study of reproductive function in persons occupationally exposed to 2,4-dichlorophenoxyacetic acid (2,4-D). Mutat Res.

[b70] Martínez-Valenzuela C, Gómez-Arroyo S, Villalobos-Pietrini R, Waliszewski S, Calderón-Segura ME, Félix-Gastélum R (2009). Genotoxic biomonitoring of agricultural workers exposed to pesticides in the north of Sinaloa State, Mexico. Environ Int.

[b71] McDuffie HH, Pahwa P, McLaughlin JR, Spinelli JJ, Fincham S, Dosman JA (2001). Non-Hodgkin's lymphoma and specific pesticide exposures in men: cross-Canada study of pesticides and health. Cancer Epidemiol Biomarkers Prev.

[b72] McDuffie HH, Pahwa P, Robson D, Dosman JA, Fincham S, Spinelli JJ (2005). Insect repellents, phenoxyherbicide exposure, and non-Hodgkin's lymphoma. J Occup Environ Med.

[b73] Miligi L, Costantini AS, Bolejack V, Veraldi A, Benvenuti A, Nanni O (2003). Non-Hodgkin's lymphoma, leukemia, and exposures in agriculture: results from the Italian multicenter case-control study. Am J Ind Med.

[b74] Miligi L, Costantini AS, Veraldi A, Benvenuti A (2006). Vineis P; WILL. *Cancer and pesticides: an overview and some results of the Italian multicenter case-control study on hematolymphopoietic malignancies*. Ann N Y Acad Sci.

[b75] Mills PK, Yang R, Riordan D (2005). Lymphohematopoietic cancers in the United Farm Workers of America (UFW), 1988–2001. Cancer Causes Control.

[b76] Mills PK, Yang R (2005). Breast cancer risk in Hispanic agricultural workers in California. Int J Occup Environ Health.

[b77] Mills PK, Yang RC (2007). Agricultural exposures and gastric cancer risk in Hispanic farm workers in California. Environ Res.

[b78] Morgan MK, Sheldon LS, Tomas KW, Egeghy PP, Croghan CW, Jones PA (2008). Adult and children's exposure to 2,4-D from multiple sources and pathways. J Expo Sci Environ Epidemiol.

[b79] Munro IC, Carlo GL, Orr JC, Sund KG, Wilson RM, Kennepohl E, Lynch BS, Jablinske M, Lee NL (1992). A comprehensive, integrated review and evaluation of the scientific evidence relating to the safety of the herbicide 2,4-D. J Am Coll Toxicol.

[b80] Pahwa P, McDuffie HH, Dosman JA, McLaughlin JR, Spinelli JJ, Robson D (2006). Hodgkin lymphoma, multiple myeloma, soft tissue sarcomas, insect repellents, and phenoxyherbicides. J Occup Environ Med.

[b81] Pahwa P, Karunanayake CP, Dosman JA, Spinelli JJ (2011). McLaughlin JR; Cross-Canada Group. *Soft-tissue sarcoma and pesticides exposure in men: results of a Canadian case-control study*. J Occup Environ Med.

[b82] Panuwet P, Prapamontol T, Chantara S, Tavornyuthikarn P, Montesano MA, Whitehead RD (2008). Concentrations of urinary pesticide metabolites in small-scale farmers in Chiang Mai Province, Tailand. Sci Total Environ.

[b83] Panuwet P, Prapamontol T, Chantara S, Barr DB (2009). Urinary pesticide metabolites in school students from northern Tailand. Int J Hyg Environ Health.

[b84] Pearce N (1989). Phenoxy herbicides and non-Hodgkin's lymphoma in New Zealand: frequency and duration of herbicide use. Br J Ind Med.

[b85] Poole C (2001). Low P-values or narrow confidence intervals: which are more durable?. Epidemiology.

[b86] Remor AP, Totti CC, Moreira DA, Dutra GP, Heuser VD, Boeira JM (2009). Occupational exposure of farm workers to pesticides: biochemical parameters and evaluation of genotoxicity. Environ Int.

[b87] Sauerhof MW, Braun WH, Blau GE, Gehring PJ (1977). The fate of 2,4-dichlorophenoxyacetic acid (2,4-D) following oral administration to man. Toxicology.

[b88] Schreinemachers DM (2003). Birth malformations and other adverse perinatal outcomes in four U.S. *Wheat-producing states*. Environ Health Perspect.

[b89] Schreinemachers DM (2010). Perturbation of lipids and glucose metabolism associated with previous 2,4-D exposure: a cross-sectional study of NHANES III data, 1988–1994. Environ Health.

[b90] Schroeder JC, Olshan AF, Baric R, Dent GA, Weinberg CR, Yount B (2001). Agricultural risk factors for t(14;18) subtypes of non-Hodgkin's lymphoma. Epidemiology.

[b91] Semchuk KM, Rosenberg AM, McDuffie HH, Cessna AJ, Pahwa P, Irvine DG (2007). Antinuclear antibodies and bromoxynil exposure in a rural sample. J Toxicol Environ Health Part A.

[b92] Slager RE, Poole JA, LeVan TD, Sandler DP, Alavanja MC, Hoppin JA (2009). Rhinitis associated with pesticide exposure among commercial pesticide applicators in the Agricultural Health Study. Occup Environ Med.

[b93] Smith JG, Christophers AJ (1992). Phenoxy herbicides and chlorophenols: a case control study on soft tissue sarcoma and malignant lymphoma. Br J Cancer.

[b94] Swaen GM, van Amelsvoort LG, Slangen JJ, Mohren DC (2004). Cancer mortality in a cohort of licensed herbicide applicators. Int Arch Occup Environ Health.

[b95] Swaen G, van Amelsvoort L (2009). A weight of evidence approach to causal inference. J Clin Epidemiol.

[b96] Swan SH, Kruse RL, Liu F, Barr DB, Drobnis EZ, Redmon JB (2003). Study for Future Families Research Group. *Semen quality in relation to biomarkers of pesticide exposure*. Environ Health Perspect.

[b97] Tanner CM, Ross GW, Jewell SA, Hauser RA, Jankovic J, Factor SA (2009). Occupation and risk of parkinsonism: a multicenter case-control study. Arch Neurol.

[b98] Tanner CM, Kamel F, Ross GW, Hoppin JA, Goldman SM, Korell M (2011). Rotenone, paraquat, and Parkinson's disease. Environ Health Perspect.

[b99] Timchalk C (2004). Comparative inter-species pharmacokinetics of phenoxyacetic acid herbicides and related organic acids *evidence that the dog is not a relevant species for evaluation of human health risk*. Toxicology.

[b100] Tomas DC (1995). Re: “When will nondifferential misclassification of an exposure preserve the direction of a trend?”. Am J Epidemiol.

[b101] Tomas KW, Dosemeci M, Hoppin JA, Sheldon LS, Croghan CW, Gordon SM (2010). Urinary biomarker, dermal, and air measurement results for 2,4-D and chlorpyrifos farm applicators in the Agricultural Health Study. J Expo Sci Environ Epidemiol.

[b102] Törn A, Gustavsson P, Sadigh J, Westerlund-Hännestrand B, Hogstedt C (2000). Mortality and cancer incidence among Swedish lumberjacks exposed to phenoxy herbicides. Occup Environ Med.

[b103] US EPA (2005). http://www.epa.gov/oppsrrd1/REDs/24d_red.pdf.

[b104] US EPA (2012). http://www.epa.gov/caddis/ssr_herb_int.html.

[b105] Valcin M, Henneberger PK, Kullman GJ, Umbach DM, London SJ, Alavanja MC (2007). Chronic bronchitis among nonsmoking farm women in the agricultural health study. J Occup Environ Med.

[b106] Waller SA, Paul K, Peterson SE, Hitti JE (2010). Agricultural-related chemical exposures, season of conception, and risk of gastroschisis in Washington State. Am J Obstet Gynecol.

[b107] Weselak M, Arbuckle TE, Wigle DT, Krewski D (2007). In utero pesticide exposure and childhood morbidity. Environ Res.

[b108] Weselak M, Arbuckle TE, Wigle DT, Walker MC, Krewski D (2008). Pre- and post-conception pesticide exposure and the risk of birth deffects in an Ontario farm population. Reprod Toxicol.

[b109] Wesseling C, van Wendel de Joode B, Monge P (2001). Pesticide-related illness and injuries among banana workers in Costa Rica: a comparison between 1993 and 1996. Int J Occup Environ Health.

[b110] Wilson RD, Geronimo J, Armbruster JA (1997). 2,4-D dissipation in field soils after applications of 2,4-D dimethylamine salt and 2,4-D 2-ethylhexyl ester. Environ Toxicol Chem.

[b111] Woods JS, Polissar L (1989). Non-Hodgkin's lymphoma among phenoxy herbicide-exposed farm workers in western Washington State. Chemosphere.

[b112] Yeary RA (1986). Urinary excretion of 2,4-D in commercial lawn specialists. Appl Ind Hyg.

[b113] Zahm SH, Weisenburger DD, Babbitt PA, Saal RC, Vaught JB, Cantor KP (1990). A case-control study of non-Hodgkin's lymphoma and the herbicide 2,4-dichlorophenoxyacetic acid (2,4-D) in eastern Nebraska. Epidemiology.

[b114] Zahm SH (1997). Mortality study of pesticide applicators and other employees of a lawn care service company. J Occup Environ Med.

[b115] Zeljezic D, Garaj-Vrhovac V (2002). Sister chromatid exchange and proliferative rate index in the longitudinal risk assessment of occupational exposure to pesticides. Chemosphere.

